# Anti-Obesity and Anti-Diabetic Effects of *Ostericum koreanum (Ganghwal)* Extract

**DOI:** 10.3390/ijms25094908

**Published:** 2024-04-30

**Authors:** Eunbi Lee, Ju-Ock Nam

**Affiliations:** 1 Department of Food Science and Biotechnology, Kyungpook National University, Daegu 41566, Republic of Korea; 21eunbi@naver.com; 2 Research Institute of Tailored Food Technology, Kyungpook National University, Daegu 41566, Republic of Korea

**Keywords:** *Ostericum koreanum*, *Ganghwal*, anti-obesity, anti-diabetes, antioxidant effect

## Abstract

“*Ganghwal*” is a widely used herbal medicine in Republic of Korea, but it has not been reported as a treatment strategy for obesity and diabetes within adipocytes. In this study, we determined that *Ostericum koreanum* extract (OKE) exerts an anti-obesity effect by inhibiting adipogenesis and an anti-diabetic effect by increasing the expression of genes related to glucose uptake in adipocytes and inhibiting α-glucosidase activity. 3T3-L1 preadipocytes were differentiated for 8 days in methylisobutylxanthine, dexamethasone, and insulin medium, and the effect of OKE was confirmed by the addition of 50 and 100 µg/mL of OKE during the differentiation process. This resulted in a reduction in lipid accumulation and the expression of PPARγ (Peroxisome proliferator-activated receptor γ) and C/EBPα (CCAAT enhancer binding protein α). Significant activation of AMPK (AMP-activated protein kinase), increased expression of GLUT4 (Glucose Transporter Type 4), and inhibition of α-glucosidase activity were also observed. These findings provide the basis for the anti-obesity and anti-diabetic effects of OKE. In addition, OKE has a significant antioxidant effect. This study presents OKE as a potential natural product-derived material for the treatment of patients with metabolic diseases such as obesity- and obesity-induced diabetes.

## 1. Introduction

As obesity increases worldwide, the number of patients with obesity has reached epidemic proportions. It was estimated that around 1 billion people worldwide will be classified as obese as of 2022, representing roughly one-eighth of the global population [1]. This trend has contributed to an increase in the prevalence of diabetes, which reached 10.5% in 2021, with an estimated 536.6 million adults aged 20 to 79 suffering from diabetes [2]. Obesity not only causes cosmetic problems but also leads to metabolic diseases such as diabetes, cardiovascular disease, and nonalcoholic fatty liver disease, all of which are major causes of death due to obesity [3]. Obesity mainly occurs when excessively consumed energy is not utilized and accumulates in the adipocytes of the body; however, genetic, physiological, environmental, psychological, and economic factors are also involved [4]. When lipids accumulate in adipose tissue, oxidative stress occurs because of lipid oxidation. In addition, monocytes are recruited to the adipose tissue, which turns into macrophages, resulting in increased secretion of inflammatory cytokines and impaired insulin signaling [5]. When insulin sensitivity decreases because of damaged insulin signaling, glucose transport to adipocytes is inhibited, causing excessive glucose to remain in the blood, which results in the development of diabetes [6]. Diabetes is also greatly affected by the activity of α-glucosidase in the small intestine. α-Glucosidase is a digestive enzyme that converts polysaccharides and disaccharides into monosaccharides in the small intestine and promotes the absorption of carbohydrates. Therefore, the use of α-glucosidase inhibitors to treat obesity-induced diabetes can regulate postprandial hyperglycemia by modulating diet-related acute glucose supply and carbohydrate digestion in the small intestine [7].

Obesity medications such as amphetamines, phentermine, and orlistat are commonly prescribed. However, these drugs have been associated with several side effects. Amphetamines and phentermine, being stimulants, may lead to serious cardiovascular events, such as cardiac arrhythmias and increased blood pressure. Orlistat, which works by inhibiting fat absorption, can cause gastrointestinal issues such as steatorrhea (fatty stools) and constipation. In rare cases, the use of these medications has also been linked to sudden death [8]. In addition, side effects such as abdominal discomfort and diarrhea have been reported with the use of existing medication for diabetes treatment [9]. Accordingly, there has been an increasing demand for new treatments because of the side effects of the currently available therapeutic drugs, which has led to the emergence of natural product-derived preparations. Natural product-derived materials are perceived to be attractive compared with pharmaceuticals because of their minimal or nonexistent side effects. In addition, the commercial market for naturally derived products, which have been researched for their efficacy and safety, is relatively large [10].

Therefore, we selected *Ostericum koreanum* as a candidate natural product-derived material for the treatment of obesity and diabetes. *Ostericum koreanum* is a widely used herbal medicine in Oriental medicine under the name “*Ganghwal*”, and has been reported to have antibacterial and anti-inflammatory effects [11,12]. Also, *O. koreanum* extract (OKE) has been reported to regulate mast cell degranulation and NO production in macrophages [13]. Before establishing this study, we considered a paper that tentatively suggests the ChondroT, a complex mixture including extract of Osterici Radix (*O. koreanum* root) along with extracts of Lonicerae Folium, Angelicae Gigantis Radix, Clematidis Radix, and Phellodendri Cortex has preventive effects against obesity and hyperlipidemia through the reduction in serum lipid levels [14]. However, there is no explanation of the mechanism in vivo nor mention of adipocytes in this paper. Health functional foods cannot be commercialized and distributed without clear mechanisms. This paper presents original research in which we identify bioactive compounds in OKE and characterize its mechanism of differentiation inhibition in adipocytes. Additionally, we present mechanisms related to anti-diabetic effects, such as increased expression of GLUT4 and adiponectin as well as a direct inhibitory effect on α-glucosidase. Based on the results of this research, OKE is proposed as a natural product-derived candidate for anti-obesity and anti-diabetic functional food material.

## 2. Results

### 2.1. OKE Is Not Cytotoxic at the Dose Used in This Experiment

**Figure 1:**
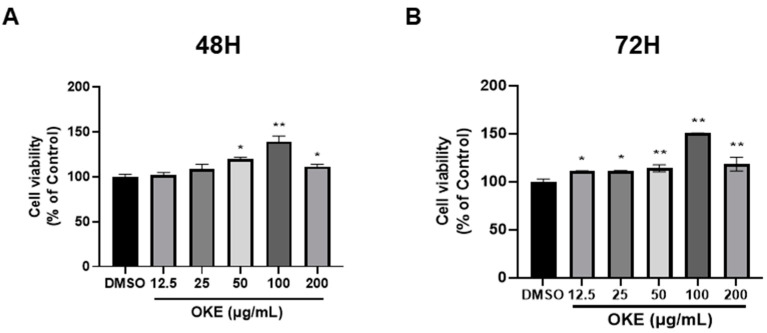
Effects of *Ostericum koreanam* extract (OKE) on the cytotoxicity of 3T3-L1 preadipocytes. To determine the dose to be used for adipogenic differentiation, 3T3-L1 preadipocytes were treated with OKE for 48 (**A**) and 72 h (**B**), and the cell viability rate was measured using the CCK assay. If the cell viability was less than 80% of the control (dimethyl sulfoxide: DMSO), the extract was considered cytotoxic. All experiments were performed in three biologically and technically independent replicates. The data are expressed as the mean ± standard deviation. * *p*  <  0.05 compared with the control (DMSO); ** *p*  <  0.01 compared with the control (DMSO).

In this section, we demonstrate that OKE did not cause toxicity within adipocytes at the doses used in this study. First, we assessed whether OKE induced cytotoxicity in 3T3-L1 preadipocytes by treating them with OKE for 48 h and 72 h and measuring cell viability using the CCK assay. OKE did not cause cytotoxicity at doses of 12.5–200 μg/mL. Cell viability significantly increased due to the OKE treatment, and this result was consistent at 48 and 72 h (Figure 1A,B). This indirectly indicates that OKE can promote cell proliferation by relieving stress occurring during the cell proliferation process. Therefore, we set the subsequent experimental dose to 50 and 100 μg/mL.

### 2.2. OKE Inhibits Adipogenesis in 3T3-L1 Adipocytes

**Figure 2:**
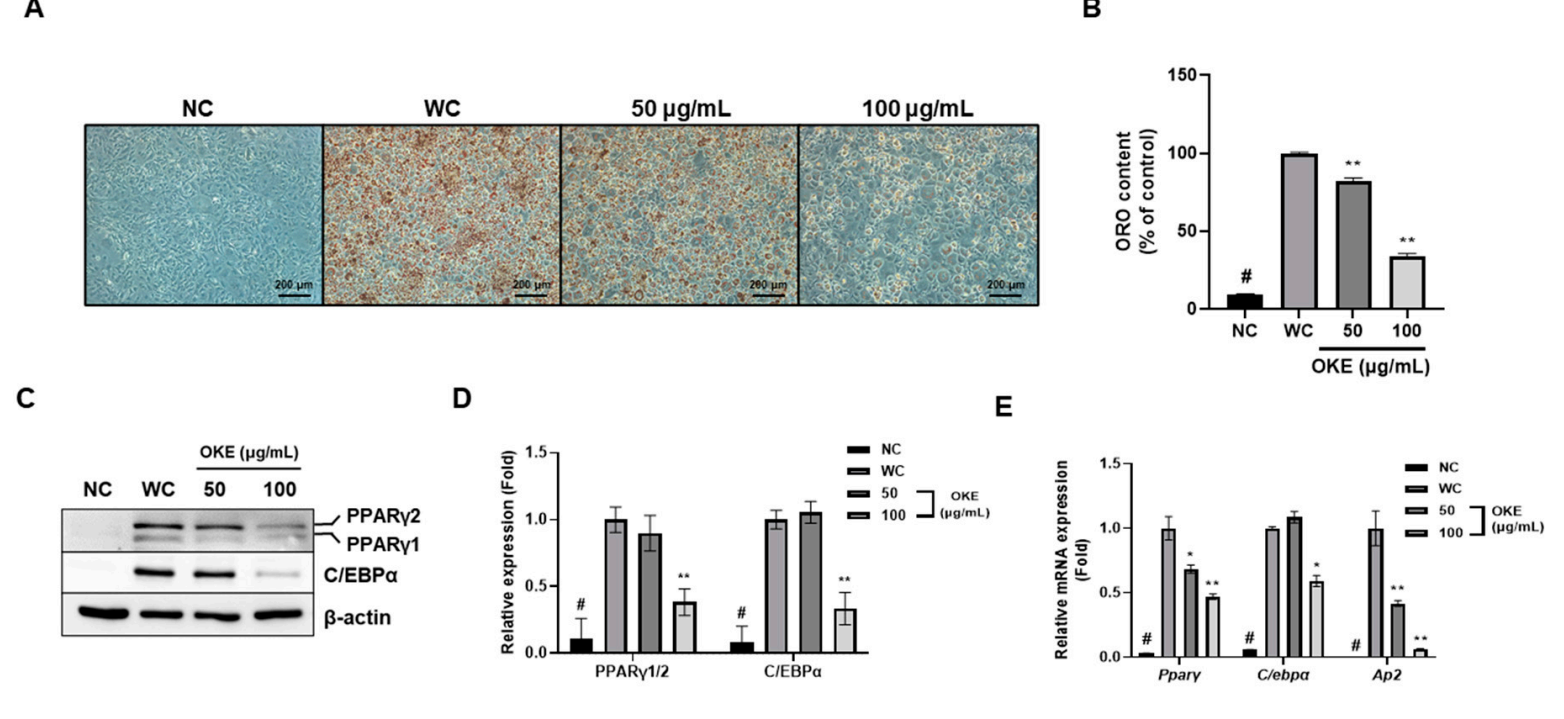
Effects of *O. koreanam* extract (OKE) on adipogenic differentiation in 3T3-L1 preadipocytes. (**A**,**B**) Mature adipocytes were stained using oil red O (ORO) staining, and ORO products were dissolved in isopropanol and quantified at 450 nm using a microplate reader. (**C**,**D**) The protein bands produced in the Western blot assay relatively indicate the expression of PPARγ1/2 and C/EBPα. The expression of β-actin was determined as the loading control, and the expression of all factors was normalized to the expression of β-actin. (**E**) The mRNA expression of *Pparγ*, *C/ebpα*, and *Ap2* in 3T3-L1 adipocytes was determined by real-time reverse transcription polymerase chain reaction (RT-PCR). The expression of all factors was normalized to the expression level of *β-actin*. All experiments were performed in three biologically and technically independent replicates. The data are expressed as the mean ± standard deviation. * *p* < 0.05 compared with the WC (White adipocytes) group; ** *p* < 0.01 compared with the WC group. # *p* < 0.01 when compared with the NC (Non-differentiated cells) group.

In this section, we discovered that OKE strongly inhibits adipogenic differentiation and lipid accumulation. To confirm the effect of OKE on adipogenic differentiation and maturation, we designed an experimental model. According to the scheme, we performed adipogenic differentiation for 8 days and formed four groups according to the presence or absence of methylisobutylxanthine, dexamethasone, and insulin (MDI) and OKE (Figure S1). Because of differentiation according to the scheme, the oil red O content decreased in a dose-dependent manner because OKE significantly inhibited adipogenesis (Figure 2A,B). These results may be supported by the decreased expression of *Pparγ* and *C/ebpα* because of the OKE treatment at the protein and mRNA levels. PPARγ and C/EBPα are critical regulators of adipogenic differentiation and lipid accumulation [15]. At the protein level, the expression of PPARγ1/2 and C/EBPα was decreased in a dose-dependent manner (Figure 2C,D); also, mRNA expression was significantly decreased (Figure 2E). In addition, the expression of *Ap2* (FABP4: fatty acid-binding protein 4), a marker of lipid accumulation in mature adipocytes [16], was also significantly decreased at the mRNA level (Figure 2E). Therefore, we suggest that the anti-obesity effect of OKE occurs through its inhibitory effects on adipogenic differentiation and lipid accumulation in adipocytes.

Additionally, to objectively analyze the anti-obesity effect of OKE, we compared it with green tea extract as a positive control. Green tea extract, which is widely used as a health functional food for reducing body fat, is known for its ability to regulate adipogenesis-related transcription factors and thus serves as a standard [17]. When comparing green tea extract at 100 μg/mL with OKE at 50 and 100 μg/mL, we found that OKE had superior inhibitory effects on adipogenesis (Figure S3A–C).

### 2.3. OKE Increases Glucose Transport-Related Gene Expression in 3T3-L1 Adipocytes

**Figure 3:**
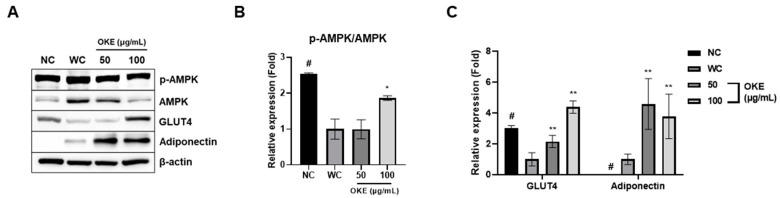
*Ostericum koreanam* extract (OKE) increases the activation of AMPK and the expression of GLUT4. (**A**,**B**) The expression levels of p-AMPK, AMPK, GLUT4, and adiponectin were expressed as protein bands by Western blotting. (**B**) The activation of AMPK was quantified by the protein bands from Western blotting. (**C**) The expression of GLUT4 and adiponectin was quantified by the protein bands from Western blotting. All experiments were performed in three biologically and technically independent replicates. The data are expressed as the mean ± standard deviation. * *p*  <  0.05 compared with the WC group; ** *p*  <  0.01 compared with the WC group. # *p* < 0.01 when compared with the NC group.

In this section, we suggest that OKE may have anti-diabetic effects in 3T3-L1 adipocytes. In the OKE 100 μg/mL group, AMPK was activated approximately twice as much as that in the WC group (Figure 3A,B). The expression of GLUT4 induced after AMPK activation also increased approximately fourfold compared with the WC group (Figure 3A,C) [18]. GLUT4 is a protein expressed in adipose tissue and is responsible for glucose uptake in response to insulin [19].

Additionally, we chose to include adiponectin, which performs a positive role in obesity patients as a representative adipokine, in the expression analysis. It was found that adiponectin expression was increased more than fourfold by the OKE treatment (Figure 3A,C). Adiponectin is an adipokine that induces glucose uptake in adipocytes and has anti-inflammatory effects [20]. Generally, in vitro expression of adiponectin has a positive correlation with adipogenic differentiation, so it is common for adiponectin expression levels to decrease when adipogenic differentiation decreases. However, OKE increased adiponectin expression while decreasing adipogenic differentiation, suggesting that OKE may simultaneously inhibit adipogenesis and improve metabolic diseases caused by obesity. Therefore, we suggest that OKE may have an anti-diabetic effect through the increased expression of GLUT4 via the activation of AMPK in adipocytes and the increased expression of adiponectin.

### 2.4. OKE Inhibits the Activity of α-Glucosidase

**Figure 4:**
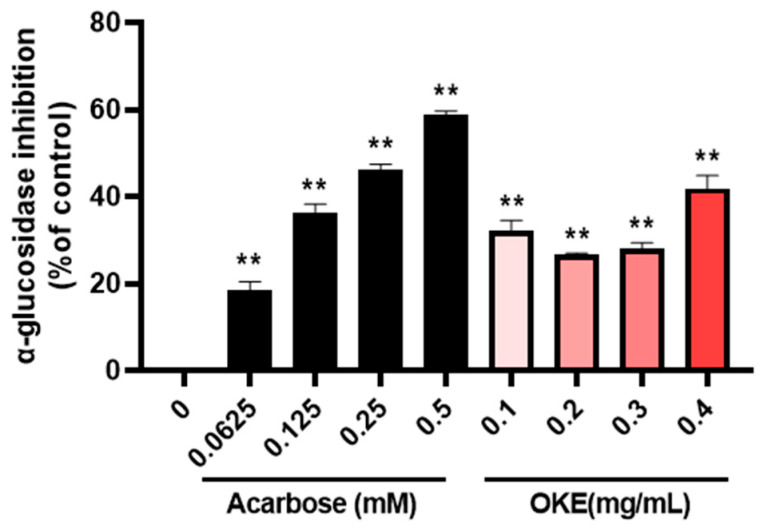
Inhibitory effect of *O. koreanam* extract (OKE) on α-glucosidase activity. OKE’s α-glucosidase inhibitory potential. Acarbose was used as the positive control, and the 0 (dimethyl sulfoxide: DMSO) group was used as the control. All experiments were performed in three biologically and technically independent replicates. The data are expressed as the mean ± standard deviation. ** *p*  <  0.01 compared with 0 group.

This section suggests that OKE may have an anti-diabetic effect by inhibiting the activity of α-glucosidase. α-Glucosidase is an important enzyme in the human body that plays a significant role in postprandial blood sugar regulation. In the epithelium of the small intestine, maltose and sucrose are hydrolyzed into glucose. Glucose is then supplied to the bloodstream, thereby raising blood sugar levels [21]. Following the observed anti-diabetic effect of OKE in adipocytes, we examined whether OKE could have an inhibitory effect on α-glucosidase activity. In this experiment, we used acarbose, a compound that has been reported as an α-glucosidase inhibitor, as the positive control [22]. Acarbose significantly inhibited α-glucosidase in a dose-dependent manner, with an inhibition rate of 58.8% at 0.5 mM. When treated with OKE, α-glucosidase was inhibited by 41% at 0.4 mg/mL (Figure 4). Based on these results, we suggest that OKE may have an anti-diabetic effect by significantly inhibiting the activity of α-glucosidase.

### 2.5. Chlorogenic Acid Is a Bioactive Compound That Allows OKE to Have Anti-Obesity and Anti-Diabetic Effects

**Figure 5:**
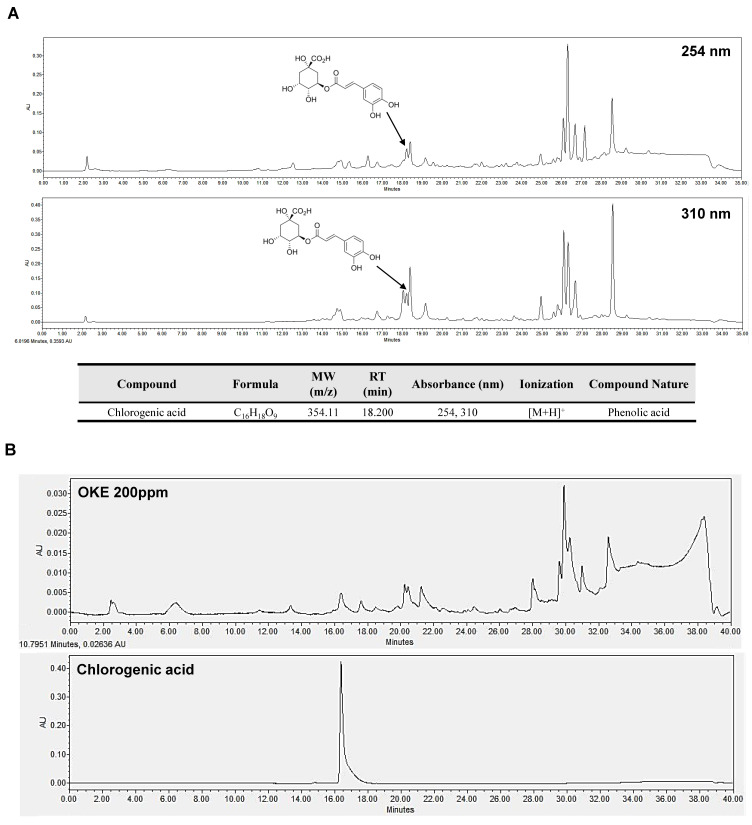
Liquid chromatography–mass spectrometry (LC-MS) and high-performance liquid chromatography (HPLC) profiling of *O. koreanam* extract (OKE). (**A**) Total ion chromatograms of OKE in the positive mode by LC-MS analysis. (**B**) Total chromatogram of OKE components and chlorogenic acid confirmed by HPLC analysis.

We performed reference-based liquid chromatography–mass spectrometry (LC-MS) and high-performance liquid chromatography (HPLC) analysis to confirm the components in OKE [23,24]. In the LC-MS analysis, 13 different peaks were detected in the positive mode analysis using absorbance at 254 and 310 nm (Figure 5A). Among these peaks, we analyzed the retention time and accurate mass in the chromatogram and confirmed that chlorogenic acid, which was previously reported as a component in “*Ganghwal*”, is present in OKE (Figure 5B) [23,24].

Chlorogenic acid has been reported to produce anti-obesity and anti-diabetic effects by inhibiting lipid synthesis and promoting glucose transport via AMPK activation [25]. Therefore, we propose chlorogenic acid as a bioactive compound with anti-obesity and anti-diabetic effects in OKE. Additionally, our results suggest that OKE contains marmesinin, nodakenin, ambroxol, and notopterol (Figure S2) [23,26,27,28].

### 2.6. Chlorogenic Acid in OKE Binds to the Active Site of α-Glucosidase to Inhibit Its Activity

**Figure 6:**
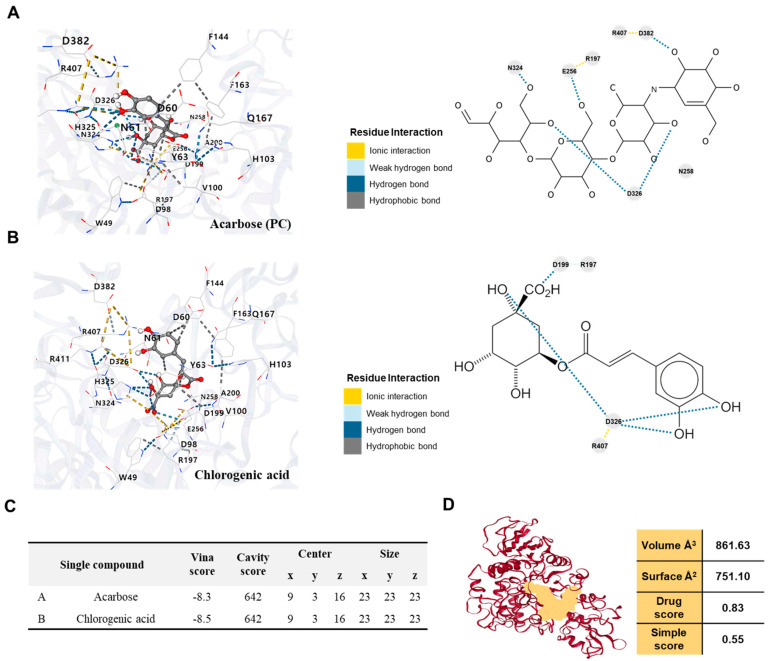
Molecular docking of α-glucosidase and *O. koreanam* extract (OKE) components. (**A**) Binding of α-glucosidase and acarbose in 3D and 2D. (**B**) Binding of α-glucosidase and chlorogenic acid in 3D and 2D. (**C**) Table providing additional information about the binding pockets in (**A**,**B**), showing the Vina score, cavity score, central coordinates, and pocket size where α-glucosidase binds to acarbose and chlorogenic acid. (**D**) Information on the pocket where acarbose, chlorogenic acid, and α-glucosidase are combined.

In this section, based on the previously confirmed potential of OKE to inhibit α-glucosidase activity, we propose that chlorogenic acid, one of the components of OKE, binds to the active site of α-glucosidase and thereby inhibits the enzyme activity. For this experiment, we selected Arg407, the active site of α-glucosidase, and Asp326, Arg197, and Asn258, the receptor sites, as targets based on a previously reported study [29]. It was then confirmed in silico whether the OKE components could bind directly or indirectly to those sites. Acarbose was able to bind directly or indirectly to the active site Arg407 and the receptor ligands Asp326, Arg197, and Asn258. Chlorogenic acid binds to Arg407, Asp326, and Arg197 (Figure 6A,B). We further confirmed that chlorogenic acid binds to the active site within the same region as the site where acarbose binds to α-glucosidase (Figure 6C). It can be seen that the binding affinity of chlorogenic acid is stronger than that of acarbose based on the results demonstrating that the absolute value of the Vina score was higher than that of acarbose (Figure 6C). In addition, the ligand pocket where these components commonly bind was found to have a volume Å^3^ of 861.63, a volume Å^2^ of 751.10, a drug score of 0.83, and a simple score of 0.55 (Figure 6D). Based on these results, we suggest that chlorogenic acid in OKE binds to the active and receptor sites of α-glucosidase and that OKE ultimately has an inhibitory effect on α-glucosidase.

### 2.7. OKE Has Antioxidant Effects

In this section, we investigate whether OKE has an antioxidant effect. The expansion of adipose tissue leads to increased levels of leptin in the bloodstream as well as the oxidation of low-density lipoprotein (LDL) cholesterol and an increase in the secretion of inflammatory cytokines, thereby triggering oxidative stress in obesity. Individuals with obesity may exist in a state of chronic oxidative stress, which has been reported as a fundamental mechanism for the onset of obesity-related complications such as diabetes [30,31]. OKE had an IC_50_ value of 125.03 µg/mL in the ABTS assay and an IC_50_ value of 242.66 in the DPPH assay. This indicates that 125.03 µg/mL of OKE is required to scavenge 50% of the radicals in the ABTS assay, and 242.66 µg/mL of OKE is required to scavenge 50% of the radicals in the DPPH assay, which suggests a significant antioxidant effect (Table 1A). In addition, the total polyphenol content in the extract was 40.64 µg/mL at OKE 400 µg/mL, and the total flavonoid content was 6.92 µg/mL at OKE 400 µg/mL. Although the content of the flavonoids in OKE was not found to be significant, the polyphenol content was significant (Table 1B). Therefore, we suggest that OKE has an antioxidant effect based on its radical scavenging ability and high polyphenol content, which suggests the potential to alleviate oxidative stress.

## 3. Discussion

In this study, two models were used to determine the anti-obesity and anti-diabetic effects of OKE. The first model indicates an inhibitory effect on differentiation and an increase in the expression of genes related to glucose uptake using the adipogenic differentiation model. The second model indicates an α-glucosidase inhibitory effect using an α-glucosidase screening model and suggests the inhibition mechanism in silico.

We used PPARγ and C/EBPα as adipogenic differentiation markers, which revealed the inhibitory effect on differentiation in the adipogenic differentiation model. PPARγ and C/EBPα are the most crucial transcription factors in adipogenic differentiation, which promote adipogenesis by coactivating the expression of adipose-specific genes, such as *AP2*, and maintaining elevated levels of their expression [32]. We propose the anti-obesity effect of OKE based on the results showing that OKE significantly reduced the expression of PPARγ, C/EBPα, and AP2 during differentiation and ultimately inhibited lipid accumulation in adipocytes. Glucose is transported intracellularly in adipocytes in an insulin-dependent manner. Increasing glucose uptake in adipocytes is a way to reduce blood glucose levels. AMPK and GLUT4 were used as markers to assess the effect of improved glucose uptake in adipocytes. When AMPK is activated, it strengthens MEF2, which leads to the increased expression of GLUT4. GLUT4 is a carrier protein that transports glucose from the outside to the inside of adipocytes. The increased expression of GLUT4 suggests an increase in glucose uptake [18]. We suggest the effect of OKE on improving glucose uptake based on the findings that OKE significantly increased the activation of AMPK and the expression of GLUT4. It is noteworthy that treatment with OKE induces the activation of AMPK in adipocytes. AMPK may promote glucose transport by activating GLUT4 but also induces the inhibition of body fat synthesis through the inhibition of adipogenic differentiation through the regulation of PPARγ and C/EBPα [33]. We suggest that both the anti-obesity and anti-diabetic effects of OKE occur via the AMPK pathway. Additionally, we showed that OKE significantly increased the expression of adiponectin, a representative adipokine. Given adiponectin’s anti-diabetic, anti-atherosclerotic, and anti-inflammatory effects, we believe that OKE may improve obesity and related metabolic diseases by reducing adipogenic differentiation and increasing adiponectin expression [34]. In this study, we aimed to characterize anti-obesity and anti-diabetic mechanisms within adipocytes, so we focused on the expression levels of adiponectin within the cells rather than the adiponectin secreted externally. As an avenue for further study, we propose evaluating the extracellular secretion of adiponectin and its levels in serum through in vivo studies. In addition, the paracrine role of adiponectin within the microenvironment could be a productive focus of future research. α-Glucosidase is a glucosidase located on the brush border of the small intestine that breaks down starch and disaccharides into glucose, thereby increasing blood glucose levels. Acarbose, a well-known α-glucosidase inhibitor, competitively and reversibly inhibits α-glucosidase in the intestine, which leads to delayed carbohydrate digestion and prolonged digestion time, thereby lowering the rate of glucose absorption into the blood [35]. Arg407 is the active site of α-glucosidase, and Asp326, Arg197, and Asn258 are known receptor sites. Acarbose binds to these positions and inhibits the activity of α-glucosidase [29]. We demonstrate that OKE has an α-glucosidase inhibitory effect and that the components of OKE directly or indirectly bind to the active site in silico.

In *O. koreanum*, also called “*Ganghwal*”, the component chlorogenic acid has been reported [23,24]. Based on these references, we confirmed the presence of chlorogenic acid within OKE through LC-MS analysis. Chlorogenic acid has been reported to inhibit adipogenic differentiation and lipid synthesis that are induced by AMPK activation [36]. In addition, it has been reported to have an α-glucosidase inhibitory effect [37]. Therefore, we propose that chlorogenic acid is the bioactive compound activating the mechanism of OKE’s anti-obesity and anti-diabetic effects presented in this paper. In addition to chlorogenic acid, OKE contains components such as marmesinin, nodakenin, ambroxol, and notopterol (Figure S2) [23,26,27,28]. Among these, nodakenin also exhibits anti-obesity activity [38], and the antioxidant effects of ambroxol [39] and the anti-inflammatory effects of notopterol [40] indirectly suggest that, beyond its direct anti-obesity effects, OKE may aid in improving complications caused by obesity. Notably, chlorogenic acid is not a particularly high-concentration component in OKE (Figure 5). We believe, however, that previous research on the mechanism of chlorogenic acid’s anti-obesity and anti-diabetic effects supports our identification of it as the major bioactive component in OKE in this regard [36,37]. Yet it is important to consider the possibility that these effects may not arise solely from chlorogenic acid but from complex synergistic interactions between chlorogenic acid and other components.

Obesity is accompanied by the expansion of adipose tissue, which results in increased levels of leptin in the bloodstream as well as the oxidation of LDL cholesterol and the secretion of inflammatory cytokines. Therefore, patients with obesity are usually in a state of chronic oxidative stress. Oxidative stress is a fundamental cause of obesity-related complications, such as diabetes [30,31]. In this study, OKE not only exhibited significant anti-obesity and anti-diabetic effects but also possessed a notable antioxidant effect. This indicates that OKE can help alleviate not only obesity but also complications caused by obesity. However, as our study focused specifically on OKE’s potential to treat obesity and diabetes, it does not provide enough experimental evidence to draw conclusions about its ability to remedy oxidative stress caused by obesity. Therefore, as a direction for future research, we propose analyzing the levels of MDA, GSH-Px, and ROS in the serum of a diet-induced obesity mouse model to evaluate the antioxidant effects of OKE and elucidate the molecular mechanisms associated with ROS scavenging.

Looking at oxidative stress from a different perspective, we confirmed that cell viability significantly increased when preadipocytes were treated with OKE (Figure 1A). We propose that this is due to the antioxidant effect of OKE. Preadipocytes are derived from mesenchymal stem cells (MSCs), and undifferentiated preadipocytes display characteristics more similar to MSCs than to adipocytes [41]. Treating MSCs with antioxidant materials can increase cell proliferation by suppressing cell death and apoptosis and reducing DNA damage caused by the oxidative stress that occurs during the proliferation process [42]. However, to support this hypothesis, additional experiments, for instance, assessing the cell cycle and apoptosis, should be conducted to establish the conditions under which proliferation may induce DNA damage due to oxidative stress. With this new information, we may be able to propose a new application for OKE.

Our study is the first to report on the mechanisms of anti-obesity and anti-diabetic effects of OKE for the discovery of health functional foods. Based on this study, utilizing *O. koreanum* as a functional health food for anti-obesity and anti-diabetic purposes could overcome the negative side effects of conventional medications, offering a safe therapeutic option for many patients with obesity and diabetes. Also, our paper aims to critique the side effects of existing anti-obesity and anti-diabetic drugs and proposes natural health functional foods that may be able to mitigate these side effects. Therefore, when considering candidate standard materials to use as a positive control for OKE, we had to choose something that can be utilized as a food ingredient, rather than a pharmaceutical. We explored using a health functional food or natural material but could not find a material that could appropriately encompass and compare with OKE’s individual anti-obesity and anti-diabetic effects and mechanisms. This is because OKE is not a single compound but an extract with multiple ingredients. Therefore, we used green tea extract, which is widely recognized and commonly used worldwide as a health functional food for reducing body fat. Green tea extract has been reported to inhibit adipogenesis-related transcription factors, thereby suppressing adipogenic differentiation and lipid accumulation, providing an anti-obesity mechanism [17]. As a result of a comparative analysis between OKE and green tea extract, we found that OKE is superior to green tea extract in inhibiting adipogenic differentiation and lipid accumulation (Figure S3).

Nevertheless, a limitation of this study is its lack of in vivo experiments that could fully verify the functional characteristics, mechanisms, biodistribution, and pharmacokinetics of OKE. As a follow-up study to this research, we propose validating the effects of OKE through preclinical experiments using a high-fat diet mouse model. We plan to focus on the anti-diabetic effects in future in vivo studies and, in particular, on the detailed mechanisms of diabetes regulation, such as the homeostasis of pancreatic β-cells and the GLP-1 pathway. Through in vivo experiments, the practical benefits of OKE’s anti-obesity and anti-diabetic effects can be verified. Additionally, providing a toxicity assessment and verifying the biodistribution and pharmacokinetics of OKE can reveal how it is processed in the body and help determine effective and safe dosages for treatment. These additional studies may contribute to the commercialization of OKE as a health functional food.

## 4. Materials and Methods

### 4.1. Preparation of OKE

*Ostericum koreanum* extract was purchased from the National Institute for Korean Medicine Development (NIKOM, Gyeongsan-si, Republic of Korea). The manufacturer prepared OKE using this protocol: First, 70% ethanol was added to 500 g of the roots of *O. koreanum* 10 times. Next, repeated 3-h reflux extractions were performed. The 70% ethanol extraction solution was then concentrated at 38 °C using a vacuum concentrator (EYELA, Tokyo, Japan). For use in subsequent experiments, the concentrated solid was diluted to an appropriate concentration using dimethyl sulfoxide (DMSO) and methanol, and impurities were removed using a 0.2 μM syringe filter (Chromdisc, Daegu, Republic of Korea).

### 4.2. Cell Culture

For the in vitro analysis, we chose the mouse preadipocyte cell line 3T3-L1 cells, which were purchased from ATCC (Manassas, VA, USA). The 3T3-L1 preadipocytes were cultured in DMEM-H (Dulbecco’s modified Eagle medium with high glucose) containing penicillin–streptomycin at 1% and NBCS (New Bovine Calf Serum) at 10% (Gibco, Paisley, UK) (*v*/*v*). The cells were grown in an incubator set at 37 °C in a humidified atmosphere containing 5% CO_2_. When 70–80% confluency was reached in a 100 mm culture dish, the cells were seeded into a 6-well plate.

### 4.3. Adipogenic Differentiation Using 3T3-L1 Mouse Preadipocytes

In a 6-well plate, preadipocytes reached 100% confluency and were maintained for an additional 2 days to reach a post-confluence state. Differentiation was then initiated by treating the cells with differentiation medium I, which consists of MDI (0.5 mM methylisobutylxanthine, 0.25 mM dexamethasone, 1 μg/mL insulin, and 0.125 nM indomethacin; Sigma, Saint Louis, MO, USA), fetal bovine serum (FBS) at 10% and penicillin–streptomycin (Gibco) at 1% (*v*/*v*). On the second day of differentiation, adipocytes were treated with differentiation medium II, which consists of 1 μg/mL of insulin, FBS (Fetal bovine serum) at 10%, and penicillin–streptomycin at 1% (Gibco) (*v*/*v*). The adipocytes were differentiated for a total of 6–8 days and then used for experiments.

### 4.4. Cell Viability Assay (CCK Assay)

To determine an appropriate concentration of OKE for treating adipocytes without cytotoxicity, cell cytotoxicity was measured using the Cell Counting Kit-8 (CCK-8; Dojindo Molecular Technologies, Kumamoto, Japan). First, 3T3-L1 cells were seeded in a 96-well plate at a density of 1 × 10^4^ cells/well and maintained for 48 h to reach confluency. Then, to evaluate the dose-dependent toxicity of the extract, the cells were treated with OKE at concentrations ranging from 12.5 to 200 µg/mL or 0.004% DMSO (control) for 48 h. To assess cell viability, 50 µL of CCK solution was added to each well, and the plate was incubated at 37 °C for 1–2 h. Data analysis was performed using a microplate reader (Tecan, Männedorf, Switzerland) at 450 nm.

### 4.5. Oil Red O Staining

At the end of adipogenic differentiation, 3T3-L1 adipocytes in a 6-well plate were washed twice with PBS (phosphate-buffered saline) to remove the medium. After washing, the adipocytes were treated with 1 mL of 4% PFA (Paraformaldehyde; Biosesang Inc., Yongin-si, Republic of Korea) and fixed for 1 h at room temperature. Then, the cells were washed twice with PBS, treated with 1 mL of 0.6% oil red O solution (Sigma), and stained for 30 min at room temperature while blocking light. After staining, the cells were washed three times with distilled water and dried for one day. To quantify the oil red O product after drying, 1 mL of isopropyl alcohol (Duksan Pure Chemicals, Ansan-si, Republic of Korea) was added to each well and stirred at 80 rpm for 10 min. The dissolved product was aliquoted at 200 μL per well in a 96-well plate and measured at 450 nm using a microplate reader.

### 4.6. Real-Time PCR

At the endpoint of adipogenic differentiation, adipocytes were washed with PBS to remove the medium. TRIzol Reagent was used to isolate total RNA (TaKaRa Bio, Kyoto, Japan) on ice. Subsequently, cDNA was synthesized using the PrimeScript^TM^ RT Reagent Kit (TaKaRa Bio). To create the cDNA library, a standard PCR (TaKaRa Bio) was performed. The thermocycler parameters included a pre-incubation step at 37 °C for 15 min, annealing at 50 °C for 5 min, extension at 98 °C for 5 min, and a final cooling step at 4 °C. The mRNA levels were measured using SYBR Green (TOYOBO, Kyoto, Japan) on an iCycler iQTM Real-Time PCR detection system (Bio-Rad Laboratories, Hercules, CA, USA). The RT-PCR thermal cycling protocol included an initial denaturation step at 95 °C, followed by 39 cycles of 15 s at 95 °C and 1 min at 60 °C. The melting phase was conducted at 95 °C for 10 s, with a final cooling step at 72.5 °C for 5 s.

The mRNA expression levels were normalized using β-actin as a reference and expressed as fold changes compared to the control group. Each experiment was conducted in triplicate, with both biological and technical replicates. Custom-designed primers for RT-PCR were provided by Macrogen (Seoul, Republic of Korea), and their sequences can be found in Table S1.

### 4.7. Western Blotting Assay

On day 8 of adipogenic differentiation (the end of differentiation), adipocytes were washed twice with PBS. Proteins were extracted from the adipocytes using RIPA buffer supplemented with 1× phosphatase and protease cocktails. The lysate was used as the protein sample after adding SDS (Biosesang Inc.) and boiling it at 100 °C for 10 min, and 20–30 μg of the protein sample was loaded on a 10–15% SDS-polyacrylamide gel for separation. The proteins were then transferred to a nitrocellulose membrane, which was incubated with TBS-T buffer (10 mM Tris pH 8.0, 150 mM NaCl, and 0.05% Tween 20) containing 5% skim milk powder for 1 h at room temperature. The membranes were incubated with the primary antibody overnight at 4 °C. After washing three times with TBS-T buffer, the membrane was incubated with horseradish peroxidase (HRP)-conjugated secondary antibody at room temperature for 1 h. Protein bands with bound antibodies were identified using an enhanced chemiluminescence kit (ECL; GE Healthcare, Buckinghamshire, UK). These bands were then visualized using the Fusion Solo Detector. To ensure accurate quantification and comparison, β-actin bands were used as normalization controls for the specific protein. Information on the antibodies used for the Western blot analysis can be found in Table S2.

### 4.8. LC-MS Analysis

OKE detection was performed using an Alliance e2695 Separations Module (Waters Corp., Milford, MA, USA) coupled with a Waters 2489 UV/Vis detector and QDa detector. Chromatographic analysis was performed on an Acquity UPLC C18 (250 mm × 3.0 mm, 5 microns) using water (solvent A) and 0.1% formic acid in methanol (Solvent B). The flow rate was 0.6 mL/min. The elution conditions were as follows: baseline equilibration for 10 min; initial conditions of 95% A, 5–25 min with 95–0% A, 25–30 min with 0% A, 30–30.01 min with 0–95% A, and 30.01–35 min with 95% A for the re-equilibration step. OKE was diluted to 10 ppm and injected at 10 μL, and chlorogenic acid was diluted to 100 ppm and injected at 5 μL. MS was operated in both the negative and positive electron spray ionization modes, with a scan range of 50–800 *m*/*z*. The probe temperature was 400 °C, and the source temperature was 120 °C. The collision energy was ramped up from 10 to 40 V, and the capillary voltages were set at 0.8 kV for the positive mode.

### 4.9. HPLC Analysis

Qualitative analysis was performed using HPLC (Waters 2695; Waters Corp.), which consists of a photodiode array (PDA) detector (Waters 2996; Waters Corp.) and a quaternary pump. When detecting chlorogenic acid, the detection wavelength was set to 191.8–400 nm. A C18 column (250 mm × 3.0 mm, 5 μM, RS Tech Corporation, Daejeon, Republic of Korea) was used as the stationary phase. Methanol (A) and water containing 0.1% trifluoroacetic acid (B) were used as the mobile phases, and the analysis was performed at a flow rate of 0.6 μL/min. The elution conditions were as follows: baseline equilibration for 10 min; initial conditions of 95% A, 5–25 min with 95–0% A, 25–30 min with 0% A, 30–30.01 min with 0–95% A, and 30.01–35 min with 95% A for the re-equilibration step. OKE was diluted to 100 ppm using methanol and injected at 20 μL, and chlorogenic acid was diluted to 10 ppm using methanol and injected at 10 μL.

### 4.10. Antioxidant Assays

#### 4.10.1. ABTS Assay

After adding 0.007 g of potassium persulfate (Sigma) to 0.0406 g of ABTS, 10 mL of distilled water was added to adjust the concentration to 7 mM, and the reagent was reacted in the dark for 12 h before being used in the experiment. After transferring 1 mL of the ABTS solution to the test tube, ascorbic acid and OKE were added at the appropriate concentration and then reacted in the dark for 30 min. The concentration of OKE was 50–400 μg/mL. Absorbance was measured at 734 nm by transferring 200 μL per sample to a 96-well plate and analyzed using a microplate reader. After setting ascorbic acid as the positive control, the IC_50_ values of ascorbic acid and OKE were measured and compared.

#### 4.10.2. DPPH Assay

A total of 0.0039 g of 2,2-diphenyl-1-picrylhydrazyl (Sigma) was dissolved in 100 mL of ethanol (Supelco, Inc., Bellefonte, PA, USA) and used in the experiment. After transferring 1 mL of DPPH solution to the test tube, ascorbic acid and OKE were added at the appropriate concentrations and reacted in the dark for 30 min. The concentration of OKE was 50–400 μg/mL. Absorbance was measured at 517 nm by transferring 200 μL per sample to a 96-well plate and analyzed using a microplate reader. After setting ascorbic acid as the positive control, the IC_50_ values of ascorbic acid and OKE were measured and compared.

#### 4.10.3. Total Polyphenol Content Assay

After making 300 μL of OKE at a concentration of 50–400 μg/mL, 250 μL of distilled water and 160 μL of 2 N Folin–Ciocalteu reagent were added, mixed, and reacted for 5 min. Next, 300 μL of 10% Na_2_CO_3_ was added and reacted in the dark for 30 min, and 200 μL was transferred to a 96-well plate. Then, the absorbance was measured at 725 nm using a microplate reader. Gallic acid was used at a concentration of 20–100 μg/mL as the standard material to calculate the standard curve.

#### 4.10.4. Total Flavonoid Content Assay

After making 0.5 mL of OKE at a concentration of 50–400 μg/mL, 0.1 mL of 10% Al(NO_3_)_3_, 0.1 mL of 1 M potassium acetate, and 4.3 mL of ethanol were sequentially added and mixed. Subsequently, they were reacted in the dark at room temperature for 40 min, and 200 μL per sample was transferred to a 96-well plate. Then, the absorbance was measured at 415 nm using a microplate reader. As a standard material to calculate the standard curve, quercetin was used at a concentration of 20–100 μg/mL.

### 4.11. α-Glucosidase Inhibition Assay

OKE was diluted to a concentration of 0.1–0.5 mg/mL with PBS. Then, 10 μL was transferred to a test tube, and 40 μL of 1 U/mL α-glucosidase (Sigma) was added to OKE and incubated at 37 °C for 10 min. Next, 80 μL of 3 mM 4-Nitrophenyl β-d-glucopyranoside (pNPG; Sigma) was added and incubated at 37 °C for 20 min. Finally, 870 μL of 0.1 M Na_2_CO_3_ (Biosesang Inc.) was added to complete the reaction, and the absorbance was measured at 405 nm using a microplate reader. As a positive control in this experiment, acarbose (Sigma) was used at a concentration range of 0.0625–0.5 mM.

### 4.12. Molecular Interaction Analysis

To confirm the binding of α-glucosidase to the components in OKE, the structure of α-glucosidase was downloaded from RSCB PDB (https://files.rcsb.org/download/2ZE0.pdb (accessed on 6 October 2023) (PDB ID: 2ZE0)). The presence of chlorogenic acid in OKE is shown in Figure 5A,B. When presenting Figure 5, we referred to Reference [23]. The structure of chlorogenic acid in OKE was obtained from PubChem (https://pubchem.ncbi.nlm.nih.gov/rest/pug/compound/CID/1794427/record/SDF?record_type=3d&response_type=save&response_basename=Conformer3D_COMPOUND_CID_1794427 (accessed on 6 October 2023)). To confirm the protein and ligand binding, the binding site center and size were calculated using CB-Dock [43], the size of the docking box was customized, and molecular docking was performed using AutoDock Vina [44].

### 4.13. Statistical Analysis

All experiments were performed in biological and technical triplicates. The results were analyzed and visualized using GraphPad Prism 9.4.1 software (San Diego, CA, USA), and all data are expressed as means ± standard deviation (SD). Statistical analyses were conducted using IBM SPSS Statistics 25. One-way ANOVAs were performed, and post hoc comparisons were made using Tukey and LSD tests, with *p*-values < 0.05 accepted as indicating statistical significance.

## 5. Conclusions

We discovered the anti-obesity effect of OKE through the inhibition of adipogenesis and the anti-diabetic effect through the increased expression of genes related to glucose uptake in adipocytes and the inhibition of α-glucosidase. To prove this, we treated adipocytes with OKE during adipogenic differentiation induced by MDI. As a result, lipid accumulation was suppressed, and the expression of key regulators of adipogenesis, such as PPARγ and C/EBPα, was decreased. In addition, the activation of AMPK and the expression of GLUT4 were increased. Both of these effects occur via the AMPK pathway, which provides strong evidence for the bioactive effects of OKE. Furthermore, the inhibition rate of α-glucosidase increased when treated with OKE, and it has been shown that chlorogenic acid, a component of OKE, binds to the active site of α-glucosidase. According to our findings, we propose chlorogenic acid as the bioactive compound in OKE with anti-obesity and anti-diabetic effects. In addition, we present the antioxidant effect of OKE, highlighting its protective effect against worsening metabolic diseases through the attenuation of obesity-induced oxidative stress. Finally, our study suggests the anti-obesity and anti-diabetic effects of OKE and suggests that OKE is a potential natural product-derived material for the treatment of patients with metabolic diseases caused by obesity.

## Figures and Tables

**Table 1:** (**A**). Antioxidant effects of *O. koreanam* extract (OKE). (**B**). Total polyphenol and flavonoid contents of *O. koreanam* extract (OKE).

(**A**)		
**Type of Assay**	**Type of Samples**	**IC_50_ (μg/mL)**
ABTS assay	Ascorbic acid (standard)	3.12 ± 0.280
	OKE	125.03 ± 1.872 **
DPPH assay	Ascorbic acid (standard)	2.83 ± 0.045
	OKE	242.66 ± 8.310 **
(**B**)		
**OKE (μg/mL)**	**TPC (GAE μg/mL)**	**TFC (μg RE/mL)**
50	13.25 ± 0.253 **	2.63 ± 0.550
100	17.30 ± 0.219 **	2.78 ± 0.476
200	24.69 ± 0.390 **	5.33 ± 1.650 **
400	40.64 ± 0.650 **	6.92 ± 0.550 **

Values are expressed as the mean ± standard deviation. All experiments were performed in triplicate. Legend for (**A**): In both experiments, the ascorbic acid group was used as the control. ** *p* < 0.01. Legend for (**B**): A reagent blank that did not contain OKE was used as the control. TPC: total polyphenol content; TFC: total flavonoid content; ** *p* < 0.01.

## Data Availability

Data are contained within the article and Supplementary Materials.

## Supplementary Materials

The supporting information can be downloaded at: https://www.mdpi.com/article/10.3390/ijms25094908/s1.
