# Supplementary material for: Anti-Obesity and Anti-Diabetic Effects of Ostericum koreanum (Ganghwal) Extract
Source: Int J Mol Sci. 2024 Apr 30;25(9):4908. doi: 10.3390/ijms25094908 (PMC11084156; doi:10.3390/ijms25094908)
Supplement: Supplementary file 1 [file ijms-25-04908-s001.zip › ijms-2956292-supplementary.pdf]

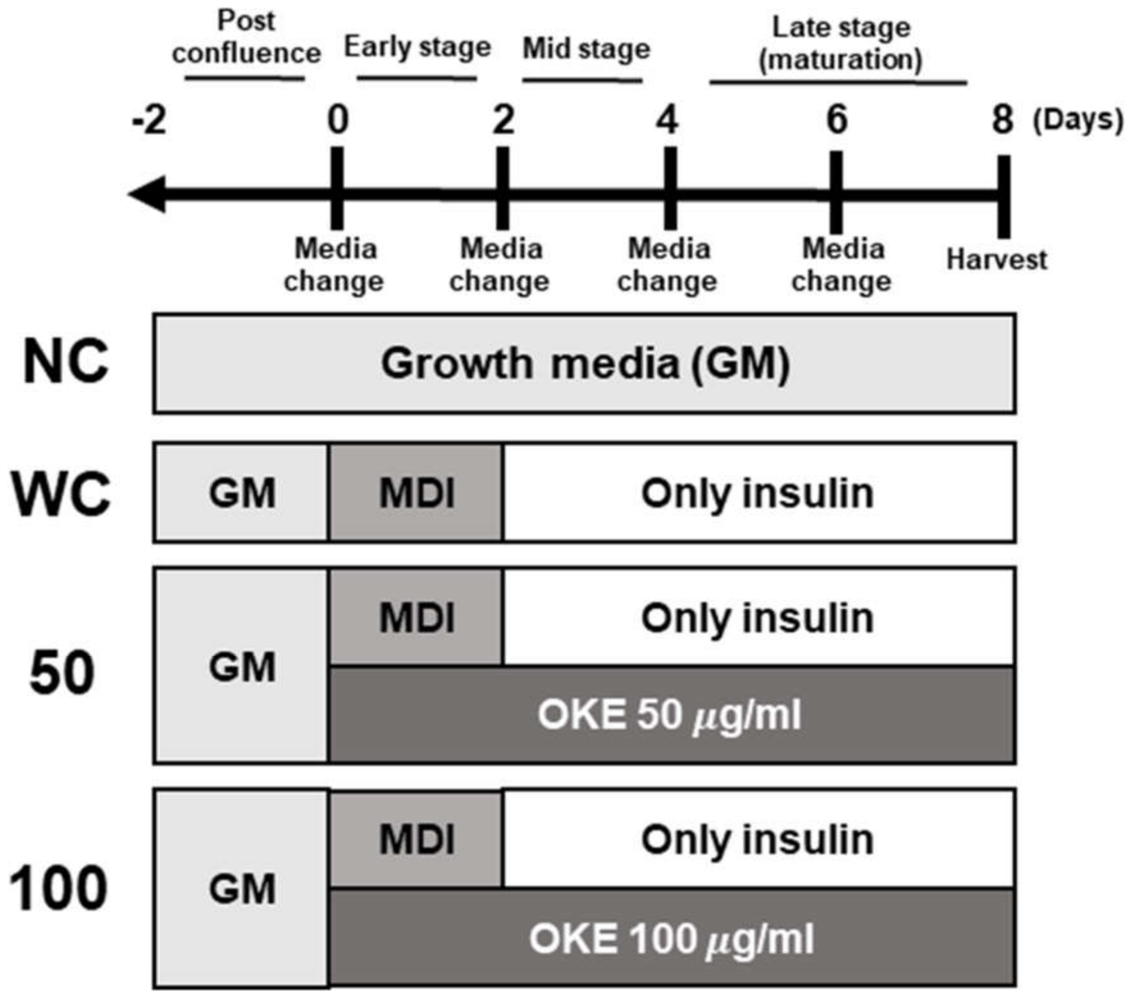


**Supplementary Figure S1.** Scheme for adipogenic differentiation of 3T3-L1 preadipocytes.

Differentiation was carried out over a total of 8 days according to the scheme, and four groups (NC; non-differentiated cell, WC; white adipocytes, 50; white adipocytes treated with 50 μg/mL OKE, 100; white adipocytes treated with 100 μg/mL) were divided according to the presence or absence of MDI and OKE.

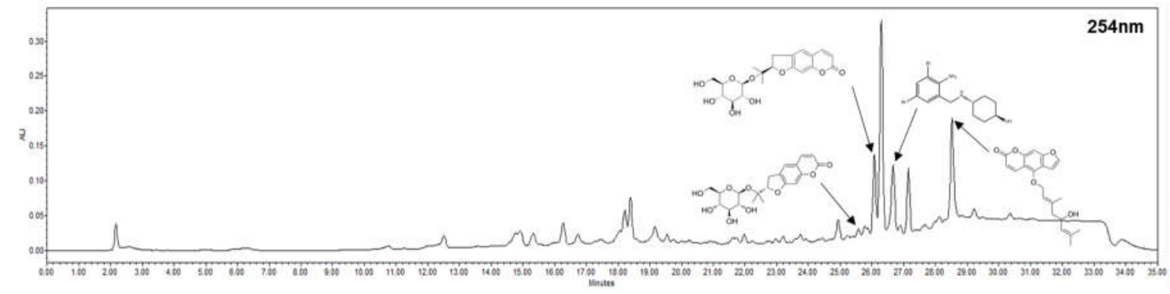


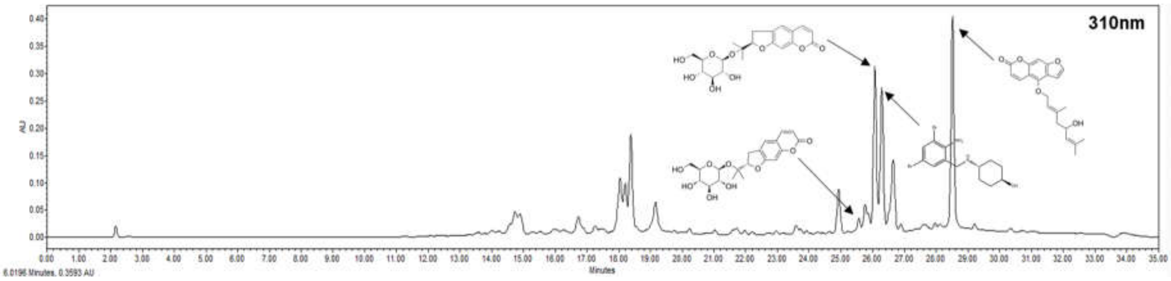


| Compound | Formula | MW (m/z) | RT (min) | Absorbance (nm) | Ionization | Compound Nature | Ref |
|---|---|---|---|---|---|---|---|
| Marmesinin | $C_{20}H_{24}O_9$ | 408.12 | 25.566 | 254, 310 | $[M+H]^+$ | Psoralen | [26] |
| Nodakenin | $C_{20}H_{24}O_9$ | 408.04 | 26.070 | 254, 310 | $[M+H]^+$ | Furanocoumarin | [23] |
| Ambroxol | $C_{13}H_{18}Br_2N_2O$ | 377.02 | 26.287 | 254, 310 | $[M+H]^+$ | Amoratic amine | [27] |
| Notopterol | $C_{21}H_{22}O_5$ | 354.44 | 28.511 | 254, 310 | $[M+H]^+$ | Furanocoumarin | [28] |

**Supplementary Figure S2**. Tentative identification of components contained in OKE

Ion chromatography of OKE in positive mode by LC-MS analysis and profiling of the tentative components.

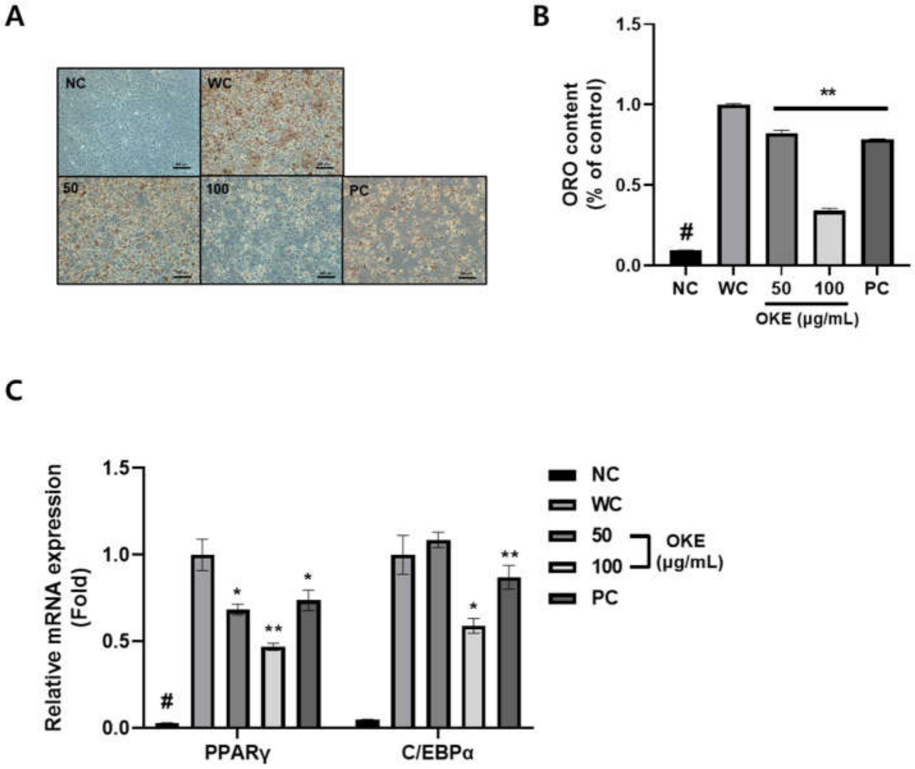


**Supplementary Figure S3**. comparison of inhibition of adipogenic differentiation and lipid accumulation between OKE and the positive control (PC).

(**A, B**) To compare the effects of OKE and PC (Green tea extract at 100 μg/mL) on inhibiting adipogenic differentiation and lipid accumulation, mature adipocytes were stained using Oil-red O staining, and Oil-red O products were dissolved in isopropanol and quantified at 450 nm using a microplate reader. (**C**) To compare the inhibitory effects of OKE and PC on adipogenic differentiation transcription factors, the mRNA expression of Pparγ and C/ebpα in 3T3-L1 adipocytes was determined by real-time reverse transcription polymerase chain reaction (RT-PCR). The expression of all factors was normalized to the expression level of β-actin. All experiments were performed in three biologically and technically independent replicates. The data are expressed as the mean ± standard deviation. * $p < 0.05$ compared with the WC group; ** $p < 0.01$ compared with the WC group. # $p < 0.01$ when compared with the NC group.

**Supplementary Table S1.** primer sequences used in RT-PCR.

| Gene name | Accession No. | | Sequence |
|---|---|---|---|
| *Ap2* | NM_001409513.1 | Forward | 5'-AAGGTGAAGAGCATAACCCT-3' |
| | | Reverse | 3'-TCACGCCTTTCATAACACATTCC-5' |
| *C/ebpα* | NM_001287523 | Forward | 5'-TTACAACAGGCCAGGTTTCC-3' |
| | | Reverse | 3'-GGCTGGCGACATACAGATCA-5' |
| *Pparγ* | AB644275 | Forward | 5'-TTTTCAAGGGTGCCAGTTTC-3' |
| | | Reverse | 3'-AATCCTTGGCCCTCTGAGAT-5' |
| *β-actin* | EF095208 | Forward | 5'-GACAACGGCTCCGGCATGTGCAAAG-3' |
| | | Reverse | 3'-TTCACGGTTGGCCTTAGGGTTCAG-5' |

**Supplementary Table S2**. **i**nformation on antibodies used in Western blot.

| Gene name | Company | Product No. | IgG | Conc. |
|---|---|---|---|---|
| **Primary antibody** | | | | |
| PPARγ | SCBT | sc-7273 | M | 1:500 |
| C/EBPα | CST | 2295S | R | 1:1000 |
| Adiponectin | Abcam | ab22554 | M | 1:1000 |
| pAMPK | CST | 2531S | R | 1:1000 |
| AMPK | CST | 2532S | R | 1:1000 |
| GLUT4 | CST | 2213S | M | 1:1000 |
| β-actin | SCBT | sc-47778 | M | 1:500 |
| **Second antibody** | | | | |
| Mouse | CST | 7076S | | 1:2000 |
| Rabbit | CST | 7074S | | 1:3000 |
